# Influence of Diabetes Mellitus and Smoking on Pro- and Anti-Inflammatory Cytokine Profiles in Gingival Crevicular Fluid

**DOI:** 10.3390/diagnostics13193051

**Published:** 2023-09-25

**Authors:** Liliana Pasarin, Maria-Alexandra Martu, Oana Elena Ciurcanu, Elena Odette Luca, Mihaela Salceanu, Diana Anton, Cristian Martu, Silvia Martu, Irina Mihaela Esanu

**Affiliations:** 1 Faculty of Dental Medicine, Grigore T. Popa University of Medicine and Pharmacy Iasi, Str. Universitatii No. 16, 700115 Iasi, Romania; liliana.pasarin@yahoo.com (L.P.); elena-odette.luca@umfiasi.ro (E.O.L.); mihaela.salceanu@umfiasi.ro (M.S.); parodontologie1@yahoo.com (S.M.); 2 Faculty of Medicine and Pharmacy, University Dunarea de Jos Galati, 35 Alexandru Ioan Cuza Street, 800010 Galati, Romania; diana_maria_a@yahoo.com; 3 Faculty of Medicine, Grigore T. Popa University of Medicine and Pharmacy Iasi, Str. Universitatii No. 16, 700115 Iasi, Romania; cristimartu@gmail.com (C.M.); irina.esanu@umfiasi.ro (I.M.E.)

**Keywords:** diabetes mellitus, smoking, pro-inflammatory cytokines, periodontal disease

## Abstract

Smoking and diabetes mellitus have been recognized as significant modifying factors of the evolution of periodontitis, being considered at the moment as descriptive factors in the periodontitis grading system. The purpose of this study was to assess the consequence of smoking, type 2 diabetes, and the combination of these two factors on clinical periodontal parameters, on the levels of gingival crevicular fluid (GCF), and also on ratios of pro-inflammatory and anti-inflammatory cytokines by using a commercially available kit-based multiplex fluorescent immunoassay. The study was carried out on 124 volunteers (control (C) group = 29, diabetes mellitus (DM) group = 32, smoking (S) group = 31, and S + DM group = 32). Total mean bleeding on probing was significantly lower in the S and S + DM groups, compared to that of the other groups (*p* < 0.05). Total amounts of TGF-β, MIP-1α, IL-6, IL-2, and IL-17 were significantly increased in the periodontally healthy sites of diabetes patients (*p* < 0.05), compared to those of the controls. Systemically healthy smoking patients had higher values of GM-CSF, TGF-β, IL-4, TNF-α, IL-5, and IL-7, while diabetic smoking patients showed higher values of IL-4, TGF-β, and MIP-1α. In smoking and systemically healthy patients, IL-23, IL-7, and IL-12 showed increased concentrations, while concentrations of TGF-β, MIP-1α, IL-2, IL-7, IL-12, IL-17, IL-21, and IL-23 were higher in smoking DM patients. In conclusion, in our study, diabetes mellitus induced a general pro-inflammatory state, while smoking mainly stimulated immunosuppression in the periodontal tissues of periodontitis subjects.

## 1. Introduction

Pro-inflammatory and anti-inflammatory cytokines exert crucial influences in the initiation and development of inflammatory and autoimmune diseases and in the host’s response to counter infection. Pathogens that are present in the periodontal pocket determine cross-signaling between multiple cell types and release cytokines that form complex networks [1,2]. The disproportion between pro-inflammatory and anti-inflammatory cytokines stimulates the progression of periodontal lesions and stops the resolution capacity of the immuno-inflammatory process [3,4,5,6].

Smoking is a risk factor for many diseases and systemic conditions, including the majority of cancers, respiratory diseases, and cardiovascular diseases. Furthermore, smoking, both active and passive, has been shown to be connected to significantly increased risks of type 2 diabetes mellitus (DM) and cardiovascular complications [7].

Although the biological mechanisms that define the relationship between smoking and diabetes may be comparable in various populations, there are important differences in smoking patterns that correlate with the particularities of a population, as well as other lifestyle behaviors. Thus, in order to establish and institute appropriate intervention strategies for the prevention and control of diabetes, it becomes essential to understand the influence that smoking has on the risk of diabetes in different populations [8].

Smoking and diabetes are recognized as significant factors that modify the evolution of periodontitis, and they are currently considered as descriptive factors in the periodontitis grading system of the current classification of periodontitis and peri-implantitis [9]. There are studies in the literature that suggest that these factors may exacerbate periodontal tissue damage through the modulation of cytokine patterns and levels [10,11,12,13,14].

The main hypothesis of our study was that although smoking and diabetes have been established as two major risk factors for periodontal destruction, they induce different individual and associated effects on the relationships between pro- and anti-inflammatory cytokines.

This study aimed to assess the effects of type 2 diabetes mellitus, smoking, and the combination of these two factors on the levels of gingival crevicular fluid (GCF) and the ratios of transforming growth factor [TGF]-β, 13 pro-inflammatory cytokines (tumor necrosis factor [TNF]-α; granulocyte-macrophage colony-stimulating factor [GM-CSF]; interferon [IFN]-γ; macrophage inflammatory protein-1α [MIP-1α]; and interleukins [IL]-1β, IL-21, IL-23, IL-2, IL-6, IL-7, IL-8, IL-12, and IL-17), and 5 anti-inflammatory cytokines (IL-4, IL-5, IL-10, and IL-13), utilizing a commercially available kit-based multiplex fluorescent immunoassay.

## 2. Materials and Methods

### 2.1. Study Design

In this cross-sectional study, carried out between September 2020 and July 2021, in the Faculty of Dental Medicine of the University of Medicine and Pharmacy “Grigore T. Popa” Iasi, Romania, subjects with periodontitis were included, sequentially selected from the population who required periodontal treatment, and assigned to one of the following groups:

- Control (C) group—non-type 2 diabetes mellitus, non-smoking individuals;
- DM group—non-smoking with type 2 diabetes mellitus individuals;
- Group S—non-type 2 diabetes mellitus smokers;
- Group S + DM—type 2 diabetes mellitus smokers.

#### 2.1.1. Inclusion Criteria

Systemically healthy patients, patients with diabetes, and patients with at least 15 teeth (excluding the third molar), with more than 35% of sites with visible plaque and/or calculus, with clinical periodontal attachment level (CAL) ≥ 4 mm and bleeding on probing (BOP), and with at least six teeth showing CAL and PD ≥ 5 mm, distributed in the four quadrants, were included in the study.

Non-diabetic patients were identified as subjects with no previous diagnosis of diabetes, fasting plasma glucose (FPG) < 99 mg/dL, and glycated hemoglobin (HbA1c) ≤ 6.0%.

Non-smoking patients were considered subjects with no history of smoking. History of consumption of ≥15 cigarettes/day (moderate to heavy smokers) for a minimum of 10 years prior (self-reported) was recorded.

The diagnosis of type 2 diabetes was established by the diabetologist, and the HbA1c values were extracted from the records of patients with diabetes.

#### 2.1.2. Exclusion Criteria

The exclusion criteria were the following: usage of antibiotics, immunosuppressive, and anti-inflammatory drugs in the past 6 months; routine use of anti-microbial mouthwashes; pregnancy; subgingival periodontal therapy in the past 6 months; other systemic inflammatory, infectious, and/or immunological diseases (e.g., osteoporosis, rheumatoid arthritis, immunological disorders, and obesity); and the main complications of diabetes (nephropathy, neuropathy, ulceration, gangrene, and amputation).

The individuals eligible for participation were briefed on the study protocol, potential discomfort, and benefits and risks, and they signed an informed consent to participate in the study.

The ethics committee for clinical research at the University of Medicine and Pharmacy “Grigore T. Popa” Iasi, Romania, approved this study protocol on 30 July 2020. The volunteers subsequently underwent periodontal treatment.

### 2.2. Periodontal Evaluation

The same calibrated examiner performed all clinical examinations in all patients. Calibration was considered acceptable if within 48 h more than 90% of the measurements were reproduced. In every tooth (excluding the third molar), the following parameters were assessed at six locations with a manual UNC15 Hu-Friedy probe: plaque index (dichotomous 0/1), bleeding on probing (BOP) (dichotomous 0/1), probing depth (PD) (in mm), and clinical attachment level (CAL) (in mm).

### 2.3. Biochemical Cytokine Assessement

Two sites with periodontal involvement (BOP present, PD and CAL ≥ 5 mm) and two healthy sites (no BOP with PD ≤ 3 mm) were chosen randomly in each individual for sampling of gingival crevicular fluid. Supragingival biofilm was removed, and the chosen sites were isolated and gently air-dried.

A strip of standard paper (Periopaper, Oraflow Inc., New York, NY, USA) was inserted approximately 2 mm into the site for 60 s. Strips contaminated with blood were discarded. The band was placed in a dry microcentrifuge tube and stored at −80 °C.

A Periotron 8000 (Oralflow Inc., NY, USA) was used to measure the volume of sampled GCF per site. The results were subsequently converted to an actual volume (μL), using as reference a pre-defined standard curve.

To each tube containing GCF, 100 μL phosphate-buffered saline was added, vortexed for 15 s, and centrifuged for 5 min (1500× *g*). The levels of various cytokines (tumor necrosis factor [TNF]-α; interferon [IFN]-γ; granulocyte-macrophage colony-stimulating factor [GM-CSF]; macrophage inflammatory protein-1α [MIP-1α]; and interleukins [IL]-1β, IL-2, IL-4, IL-5, IL-6, IL-7, IL-8, IL-10, IL-12, IL-13, IL-17, IL-21, and IL-23) and the transforming growth factor [TGF]-β were assessed using a commercially available kit-based multiplex fluorescent immunoassay (Milliplex^®^ MAP—High Sensitivity Human T Cells and TGF-β1 single Plex, EMD Millipore Corporation, Burlington, MA, USA) and a plate reader (Magpix^®^, EMD Millipore Corporation).

The protein amounts in each sample were estimated using the appropriate software (Milliplex^®^ Analyst 5.1, EMD Millipore).

Samples that recorded protein levels under the detection limit of the assay were reported as 0. All analyses were performed “blind”.

All procedures were performed according to the manufacturer’s recommendations.

The results were described as total amount (pg/site) and concentration (pg/μL).

### 2.4. Statistical Analysis

All data were recorded in individual patient records, stored, and statistically analyzed. For the statistical analysis, we used Microsoft Excel 2021 (Microsoft, Redmond, WA, USA) and Wizard 2 for Mac (Evan Miller^®^) software.

The Shapiro–Wilk test was performed to determine the normality of data distribution. Normally distributed values were compared with the paired *t*-test, and for non-normally distributed values, we used the Mann–Whitney test.

The level of significance was set at *p* < 0.05. The Kruskal–Wallis and Dunn’s post-hoc tests were used for multiple comparison analysis between groups.

## 3. Results

### 3.1. Demographic and Clinical Parameters

The study was carried out on 124 individuals (C group = 29, DM group = 32, S group = 31, and S + DM group = 32), aged between 31 and 74 years. The groups did not have statistically significant differences in terms of gender and age (*p* > 0.05).

The total mean bleeding on probing (in the oral cavity) was significantly decreased in the S and S + DM groups versus that in the other groups (*p* < 0.05). Higher HbA1c levels were observed in the DM and S + DM groups than in the control and S groups (*p* < 0.05; Table 1).

Probing depths and attachment loss were significantly greater for the DM and S + DM groups, with the highest values for smoking patients with diabetes.

All subjects with diabetes reported using metformin and followed a controlled diet. Eight and five subjects in the S + DM and DM groups, respectively, stated also using insulin. Diabetes treatment types did not significantly differ between DM and S + DM groups (*p* > 0.05).

### 3.2. Cytokine Values

The total amounts of MIP-1α, IL-2, IL-6, IL-17, and TGF-β were significantly increased in the non-diseased sites of diabetes patients (*p* < 0.05), compared to those in the control group. In addition, the total amounts of TGF-β were significantly higher in periodontally healthy sites in both systemically healthy and diabetic smoking patients, compared to those in the controls (*p* < 0.05) (Table 2).

In periodontally affected sites, non-smoking diabetic patients showed significantly higher values than those in the control group for most cytokines, except for TNF-α, IL-4, IL-5, IL-7, and GM-CSF (Table 3). Systemically healthy smoking patients had higher values of GM-CSF, TNF-α, TGF-β, IL-4, IL-5, and IL-7, while diabetic smoking patients showed higher values of IL-4, TGF-β, and MIP-1α (Table 3).

In the periodontally healthy sites, the concentrations of IL-4, IL-5, IL-7, and IL-13 were decreased in the diabetes group compared to those in the control group; TGF-β concentrations were also decreased in the control group versus those in the rest of the groups (*p* < 0.05). In the periodontally affected sites, in non-smoking patients with DM, the concentrations of IL-2, IL-6, IL-17, IL-21, TGF-β, and MIP-1α were increased when compared to those in the controls (*p* < 0 0.05) (Table 4).

In smoking and systemically healthy patients, IL-7, IL-12, and IL-23 showed increased concentrations, while concentrations of IL-2, IL-7, IL-12, IL-17, IL-21, IL-23, MIP-1α, and TGF-β were higher in smoking DM patients (*p* < 0.05) (Table 5).

**Figure 1:**
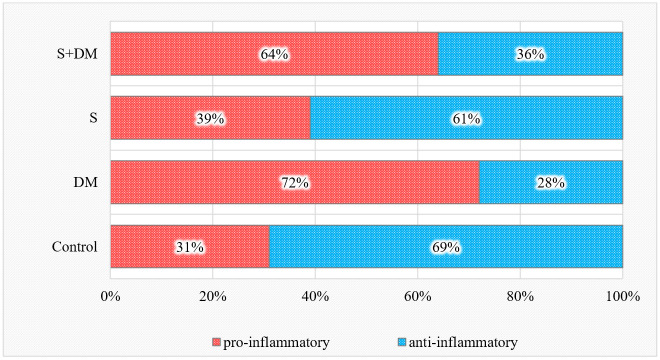
Total pro- and anti-inflammatory cytokine values.

Considering both healthy and diseased sites, the total distribution of anti- and pro-inflammatory cytokines in relation to all of the studied cytokines is illustrated in Figure 1.

The proportion of pro-inflammatory cytokines was elevated in the diabetic non-smoking group and decreased in the smoking group. At the same time, the ratio of anti-inflammatory cytokines in relation to all 18 studied cytokines was increased in smokers and decreased in both diabetic groups versus those in the controls (*p* < 0.05).

## 4. Discussion

This cross-sectional study proposed an evaluation of the effects of type 2 diabetes mellitus, smoking, and these two factors combined on the inflammatory status in crevicular fluid. Although there are numerous studies in the specialized literature that have focused on the individual repercussions of smoking and diabetes on cytokine concentrations in periodontal tissues, the association of these two highly modulatory factors remains incompletely elucidated.

The results of our research demonstrated that the presence of diabetes affected the proportions of pro- and anti-inflammatory cytokines, stimulating the general proportions of pro-inflammatory cytokines and inhibiting anti-inflammatory cytokines, illustrating that diabetes mellitus generated a pro-inflammatory state.

Diabetes mellitus increased the concentrations of pro-inflammatory cytokines in non-smoking patients, whether or not the sites were periodontally affected; decreased anti-inflammatory cytokines; and generated higher pro- and anti-inflammatory cytokine ratios. Those individuals also showed elevated proportions of pro-inflammatory cytokines and reduced proportions of anti-inflammatory cytokines compared to those of the controls.

The results from our study indicated that diabetes mellitus leads to a general pro-inflammatory condition in periodontal pockets, which was also observed in previous investigations [10,11,15].

Hyperglycemic status in periodontal tissues could induce oxidative stress and the generation of advanced glycation end products (AGEs), thus explaining the pro-inflammatory state of diabetic subjects [16]. The binding of AGEs to RAGE activates a cascade of cell signaling events, resulting in the overproduction of pro-inflammatory cytokines [17]. Hyperglycemic status and AGEs generate reactive oxygen species, causing continuous production of AGEs and accumulation of pro-inflammatory cytokines. In addition, an increase in pro-inflammatory cytokines levels favors the formation of free radicals [18].

According to our results, smoking in turn altered pro-/anti-inflammatory cytokine ratios, especially in periodontally affected sites, causing a decrease in the pro-inflammatory cytokine proportion, highlighting that smoking primarily increased immunosuppression in periodontal pockets. Diabetes mellitus smoking patients, however, did not illustrate the immunosuppressed response seen in smokers or the hyper-inflammatory response observed in diabetics, displaying a compelling counterbalance from the combination of these two risk factors. These data support the premise that diabetes mellitus and/or smoking generate significant influences on cytokines, but in distinct ways. It is suggested that changes in the balance of the cytokine networks caused by these risk factors to skew towards the activation or suppression of inflammation could generate negative consequences on periodontal tissues.

From the data obtained in our research, smoking appears to stimulate immunosuppression in periodontal pockets instead of an inflammatory status, especially during the periodontal pathogens’ response. In samples collected from smoking patients, we observed lower levels of pro- and anti-inflammatory molecules and elevated proportions of anti-inflammatory cytokines when compared to those in the controls. Supporting these results, past studies noted the role of smoking in the reduced manifestation of inflammatory signs [15,19,20].

Studies in the literature aimed at smoking behavior, albeit the link between diabetes mellitus risk and other tobacco use behaviors was less accounted for. In a joint analysis of five cohort studies, the increased use of smokeless tobacco was highlighted as a significant risk factor for type 2 diabetes. The authors recognized a similar risk as that of cigarette smoking, indicating that heated tobacco devices are not a beneficial alternative in reducing the risk of type 2 diabetes [21]. E-cigarettes have grown in popularity; however, their repercussions on health, especially diabetes, need further investigation [22].

In a cohort study of 53,930 subjects (healthcare workers) and 2441 incident cases of type 2 diabetes during a median follow-up of 4 years, the adjusted risk ratio (95% CI) for diabetes was 1.16 (1.04–1.30) for ex-smokers and 1.34 (1.22–1.48) for current smokers, compared to that of never-smokers after adjusting for sex, age, job, BMI, hypertension, and waist circumference [23]. Comparable observations were detected in another study, with a follow-up period of 12 years on a group of 7543 individuals. The corresponding adjusted risk ratios were 1.18 (1.02–1.36) and 1.61 (1.42–1.83), respectively, after adjusting for lifestyle and other metabolical risk factors [24].

The link between diabetes and passive smoking has also been investigated; in a systematic review, exposure to secondhand smoke was found to be linked with a 22% increased risk of type 2 diabetes mellitus [25]. The data presented provide strong evidence to support anti-smoking policies, with the aim of protecting non-smokers from the detrimental outcomes of passive smoking. In addition, the effects of passive smoking on patients with type 2 diabetes should also be highlighted for diabetes prevention and control on a larger population scale.

One of the main causes of mortality among patients with diabetes is macrovascular complications (for example, atherosclerosis); they are also the biggest factor contributing to the costs of diabetes, both direct and indirect [26]. Smoking is a well-documented risk factor for cardiovascular disease in the general population, and the literature studies demonstrate that this habit elevates macrovascular complications risk in diabetic subjects [1].

An analysis by Zhu et al. (2017) revealed that smoking was linked to an approximately 50% high-end risk of cardiovascular complications in subjects with type 2 diabetes, with the highest point suppressants for peripheral arterial disease (combined risk ratio 2.15; 95% CI 1.62–2.85). In comparison to non-smokers, ex-smokers also had a moderate 10–20% risk of mortality by chronic cardiovascular disease, but not stroke [27]. Several large studies support these observations. In a large retrospective cohort study of 132,462 patients with type 2 diabetes mellitus, it was observed that smoking was linked with an increased risk of mortality in both sexes during the follow-up period of 5 years [28].

Microvascular complications mainly include retinopathy, nephropathy, and neuropathy; these complications may be caused by hyperglycemic injury to small blood vessels. The correlations between microangiopathies and smoking have been investigated in numerous studies; however, the results are inconclusive [29].

Researchers have tried to clarify the connection between smoking and diabetes through several mechanisms. Tobacco and nicotine are appetite suppressants via signaling in the hypothalamus, resulting in a diminished intake of food and energy consumption through direct and indirect effects, thus decreasing body weight. Nevertheless, smoking heightens obesity risk via an anti-estrogenic effect; moreover, abdominal obesity is greatly associated with diabetes and insulin resistance [30].

Nicotine also stimulates lipolysis and free fatty acids’ release in the liver and skeletal muscles; these phenomena are linked to the heightened hepatic secretion of very low-density lipoproteins and intracellular lipid saturation, along with insulin resistance. Moreover, inflammation and oxidative stress is increased through smoking, hence diminishing endothelial function, leading to insulin resistance and diabetes as well as chronic complications such as diabetic nephropathy or periodontal disease [8]. In addition, smoking affects functional nicotinic receptors on beta cells and pancreatic islets, negatively influencing insulin release [31].

An important observation in our study was that the diabetes-induced pro-inflammatory status in periodontal tissues was somewhat compensated by smoking. Diabetic smokers illustrated a trend towards a pro-inflammatory profile in both healthy and diseased periodontal sites, though in a smaller amount when compared to those of non-smoking diabetic patients.

Another significant fact was that individual TGF-β levels were increased, while the ratios of various pro-inflammatory cytokines and TGF-β were reduced in the periodontal sites of all risk factor groups. TGF-β is a molecule with anti-inflammatory/immunosuppressive action; increased levels of this molecule in diabetic patients may signify an unsuccessful effort by the host to manage its pro-inflammatory state, whereas in smoking subjects, elevated levels of TGF-β appeared to be partially responsible for prolonging immunosuppression.

It is intriguing to compare these results regarding the repercussions of diabetes and smoking on pro- and anti-inflammatory cytokines released locally with data published previously concerning the influence of the same risk factors on circulatory ratios of pro- and anti-inflammatory cytokines [32].

Previous studies have revealed that periodontal disease, when coupled with smoking and/or diabetes, is correlated to the global systemic pro-inflammatory load. Thus, it is hypothesized that smoking and diabetes in individuals with periodontitis modifies the local periodontal and circulating cytokine profiles in distinct pathways [33].

A single cytokine molecule may generate limited activity during the host’s inflammatory response to periodontal pathogens; its effect, however, is potentiated by its interaction with other cytokines. It has been deliberated that this cytokine clustering pattern and the elevated individual proportions of pro- and anti-inflammatory cytokines noticed in healthy sites of individuals with diabetes mellitus may conduce to placing these sites in the risk zone for periodontal disease [34]. Moreover, these complex cytokine networks should be explored in other contexts of periodontitis and other systemic diseases and conditions and periodontal disease treatments [35,36,37,38,39,40].

We also observed that six molecules (IL-4, IL-5, IL-12, IL-13, IL-21, and IL-23) were intimately related in the periodontitis sites of all evaluated groups, regardless of risk factors. Thus, diabetes mellitus appears to significantly influence cytokine interactions in healthy sites; however, with the presence of periodontal involvement, cytokine relationships were influenced by periodontal infection rather than risk factors.

Hence, these results do not in any way support that the association between diabetes mellitus and smoking generates a more protective or favorable inflammatory profile; the clinical ramifications of such a cytokine profile in diabetes mellitus smokers are uncharted. Moreover, besides the studied cytokines, a number of other factors could be implicated in the periodontal destructive inflammatory phenomena in diabetic smoking patients. Further investigations that focus on the combined effects of diabetes and smoking on the etiopathogenesis of periodontitis are necessary.

To our knowledge, our study is the first to propose this direct parallel between the effect of diabetes, smoking, and the combination of both risk factors on the levels and proportions of eighteen cytokines in the healthy and diseased sites of periodontitis patients.

An important limitation of this study is its cross-sectional nature. Thus, a causal relationship cannot be established between variables. With that said, our study may provide evidence for the association between cytokines and the studied risk factors. In addition, the smoking status of the individuals was self-reported and thus subjective, based on the subjects’ memory. For an objective investigation, the evaluation of nicotine in urine or serum would be more useful as an indicator of smoking status. Moreover, the multiplex assay used detected cytokines released by whole cell populations. Pertinent observations could be derived from the characterization of cytokine profiles at the level of specific cells by technologies such as intracellular flow cytometry.

In the future, we plan to further explore the intricate networks that govern the pro- and anti-inflammatory response of patients that have multiple risk factors, such as smoking, diabetes, hypertension, autoimmune diseases, and other diseases and conditions, along with medication that may influence local and systemic molecular pathways. Furthermore, as smokeless tobacco is becoming more popular, there needs to be larger, longitudinal, and cross-sectional studies that assess the local and systemic effects of this type of habit on the overall health of the patient.

Smoking cessation and control interventions both at an individual level and at the population level are practical for diminishing the health damage that smoking causes. Multiple measures have been instituted, such as an increased tobacco tax, bans on advertising that promotes smoking, bans on smoking in enclosed spaces, the use of discouraging images on cigarette packs, etc.

Even though smoking cessation is recommended by the American Diabetes Association as a standard component of diabetes treatments and it endorses counseling and other forms of smoking cessation treatment, there are insufficient data on the validity of pharmacological interventions and behavioral therapies for smoking cessation in diabetic individuals [41].

Although diabetes and smoking are established risk factors for periodontal destruction, they show different ways of influencing the cytokine profile underlying the destruction. In the concurrent presence of the two factors, smoking appears to have partially attenuated the hyperinflammatory effect of diabetes.

In our study, the association between diabetes mellitus and smoking does not produce a more protective or favorable inflammatory profile, and the clinical ramifications of such a cytokine profile in diabetic smokers are unknown. Our study draws attention to the need for urgent attitudes and actions for smoking cessation as an essential component in the prevention and management of diabetes mellitus, especially in patients with periodontal disease.

## 5. Conclusions

In our study, the proportion of pro-inflammatory cytokines was higher in non-smoking patients with diabetes and lower in smoking individuals. The proportion of anti-inflammatory cytokines was decreased in both diabetic groups and elevated in the smoking group versus in the controls. Diabetes mellitus caused a general pro-inflammatory state, while smoking mainly stimulated immunosuppression in the periodontal tissues of periodontitis subjects.

## Figures and Tables

**Table 1:** Clinical and demographic parameters.

Parameter		Group				*p*
		C (*n* = 29)	DM (*n* = 32)	S (*n* = 31)	S + DM (*n* = 32)	
Sex	M (*n*; %)	16; 55.17	17; 53.12	18; 58.06	16; 50.00	0.97
	F (*n*; %)	13; 44.83	15; 46.88	13; 41.94	16; 50.00	
Age (years)		50.3 ± 7.2	54.6 ± 8.1	52.7 ± 8.6	51.8 ± 7.9	0.68
Years smoking		-	-	19.4 ± 2.5	17.9 ± 2.2	0.27
Nr. of cigarettes/day		-	-	13.6 ± 3.4	12.4 ± 2.9	0.84
Years DM		-	9.4 ± 3.5	-	8.5 ± 2.8	0.12
HbA1c (%)		5.6 ± 0.6 A	7.6 ± 0.4 B	5.9 ± 0.5 A	7.8 ± 0.7 B	0.001
Sites with dental plaque (%) in the oral cavity		61.5 ± 22.4	62.4 ± 21.2	67.9 ± 23.7	64.3 ± 24.6	0.47
Sites with BOP (%) in the oral cavity		42.8 ± 16.3 A	66.3 ± 21.4 A	21.5 ± 9.6 B	44.7 ± 17.3 B	0.002
PD (mm) in the oral cavity		3.3 ± 0.7 A	4.5 ± 1.2 A	4.4 ± 1.2 A	4.9 ± 1.8 A	0.02
CAL (mm) in the oral cavity		1.6 ± 0.5 A	2.2 ± 0.8	2.4 ± 1.1 A	3.1 ± 1.5 A	0.01
PD (mm) per healthy site		1.8 ± 0.6 A	2.1 ± 0.7	2.4 ± 0.8 A	2.9 ± 1.1 A	0.03
PD (mm) per diseased site		5.4 ± 2.1 A	6.2 ± 2.4 B	6.1 ± 2.3 A	8.5 ± 3.7 AB	0.001
CAL (mm) per diseased site		3.1 ± 1.3	3.8 ± 1.6 A	3.6 ± 1.5	4.7 ± 2.1 A	0.008

Different letters express variations among groups by Tukey’s test and one-way ANOVA (*p* < 0.05). All data other than sex are illustrated as mean ± SD (standard deviation). Abbreviations: C = non-diabetes mellitus, non-smoking individuals; DM = type 2 diabetes mellitus non-smoking individuals; S = smoking individuals without diabetes mellitus; S + DM = smoking individuals with type 2 diabetes mellitus; PD = probing depth; CAL = clinical attachment level; BOP = bleeding on probing; HbA1c = glycated hemoglobin; M = male; and F = female.

**Table 2:** Total cytokine values in healthy sites.

	Group			
	C (*n* = 29)	DM (*n* = 32)	S (*n* = 31)	S + DM (*n* = 32)
IL-2	4.6 ± 1.2	5.8 ± 2.1 *	5.1 ± 2.2	4.8 ± 1.7
IL-6	2.1 ± 0.8	4.3 ± 2.0 *	2.0 ± 0.4	2.5 ± 0.9
IL-17	2.6 ± 0.8	3.7 ± 1.1 *	2.7 ± 0.9	2.9 ± 1.1
TGF-β	5.2 ± 4.2	13.8 ± 9.7 *	18.5 ± 10.4 *	12.3 ± 10.8 *
MIP-1 α	8.9 ± 7.2	14.2 ± 10.7 *	6.3 ± 6.4	6.9 ± 6.3

* Significantly different from the control group (*p* < 0.05). C = non-diabetes mellitus, non-smoking individuals; DM = type 2 diabetes mellitus non-smoking individuals; S = smoking individuals without diabetes mellitus; S + DM = smoking individuals with type 2 diabetes mellitus.

**Table 3:** Total cytokine values in periodontally affected sites.

	Group			
	C (*n* = 29)	DM (*n* = 32)	S (*n* = 31)	S + DM (*n* = 32)
IL-2	5.6 ± 1.1	8.9 ± 2.4 *	5.7 ± 1.6	5.4 ± 1.4
IL-4	1.2 ± 0.2	1.1 ± 0.1 *	1.0 ± 0.1 *	1.0 ± 0.2 *
IL-5	1.1 ± 0.2	1.0 ± 0.1	0.7 ± 0.3 *	1.0 ± 0.1
IL-6	2.3 ± 0.5	7.8 ± 8.1 *	3.7 ± 3.8	2.1 ± 1.8
IL-7	3.6 ± 1.7	3.8 ± 2.1	2.2 ± 1.6 *	3.1 ± 1.5
IL-17	2.9 ± 0.9	5.2 ± 2.5 *	3.1 ± 2.3	3.0 ± 2.8
IL-21	1.3 ± 1.1	1.9 ± 0.9 *	1.1 ± 0.8	1.2 ± 0.5
TNF-α	9.1 ± 1.6	7.3 ± 2.9	4.1 ± 4.7 *	6.7 ± 5.1
TGF-β	5.9 ± 2.7	19.1 ± 8.8 *	33.6 ± 11.5 *	58.2 ± 12.2 *
MIP-1α	9.4 ± 2.7	24.1 ± 10.1 *	10.2 ± 8.2	13.5 ± 8.6 *
GM-CSF	5.7 ± 2.7	6.1 ± 2.8	3.4 ± 1.5 *	4.8 ± 1.9

* Significantly different from the control group using the Kruskal–Wallis test and Dunn’s test (*p* < 0.05). C = non-diabetes mellitus, non-smoking individuals; DM = type 2 diabetes mellitus non-smoking individuals; S = smoking individuals without diabetes mellitus; S + DM = smoking individuals with type 2 diabetes mellitus.

**Table 4:** Total cytokine values in healthy sites.

	Group			
	C (*n* = 29)	DM (*n* = 32)	S (*n* = 31)	S + DM (*n* = 32)
IL-4	12.1 ± 12.9	6.3 ± 6.4 *	10.2 ± 9.7	12.2 ± 16.2
IL-5	10.3 ± 12.1	6.4 ± 6.9 *	9.2 ± 11.2	11.1 ± 16.8
IL-7	28.5 ± 34.7	14.2 ± 17.3 *	20.1 ± 26.9	31.7 ± 34.8
IL-13	16.6 ± 21.5	7.2 ± 9.4 *	14.1 ± 16.1	17.4 ± 24.2
TGF-β	42.6 ± 54.2	58.9 ± 61.7 *	114.7 ± 132.8 *	101.4 ± 156.9 *

* Significantly different from the control group (*p* < 0.05). C = non-diabetes mellitus, non-smoking individuals; DM = type 2 diabetes mellitus non-smoking individuals; S = smoking individuals without diabetes mellitus; S + DM = smoking individuals with type 2 diabetes mellitus.

**Table 5:** Total cytokine values in periodontally affected sites.

	Group			
	C (*n* = 29)	DM (*n* = 32)	S (*n* = 31)	S + DM (*n* = 32)
IL-2	0.8 ± 1.1	1.1 ± 1.4 *	1.1 ± 1.3 *	1.3 ± 1.4 *
IL-6	4.5 ± 4.8	8.1 ± 9.4 *	5.2 ± 10.3	5.1 ± 6.8
IL-7	5.7 ± 7.1	6.8 ± 6.9	7.1 ± 8.5 *	9.1 ± 10.1 *
IL-12	18.2 ± 18.6	19.6 ± 20.5	22.6 ± 31.5	29.3 ± 32.4 *
IL-17	5.3 ± 7.1	7.6 ± 8.1 *	6.3 ± 6.9	9.1 ± 11.3 *
IL-21	2.1 ± 3.6	3.2 ± 3.74.6 *	2.9 ± 3.9	4.2 ± 4.7 *
IL-23	5.1 ± 7.5	5.6 ± 9.9	5.7 ± 6.4	8.1 ± 8.9 *
TGF-β	7.2 ± 8.3	52.7 ± 64.7 *	48.3 ± 53.2 *	36.4 ± 37.2 *
MIP-1α	12.1 ± 14.2	27.4 ± 32.3 *	14.5 ± 15.6	19.3 ± 25.6

* Significantly different from the control group (*p* < 0.05). C = non-diabetes mellitus, non-smoking individuals; DM = type 2 diabetes mellitus non-smoking individuals; S = smoking individuals without diabetes mellitus; S + DM = smoking individuals with type 2 diabetes mellitus.

## Data Availability

The data used to support the findings of this study are available from the corresponding author upon reasonable request.
